# Characterization of HIV-Associated Neurocognitive Impairment in Middle-Aged and Older Persons With HIV in Lima, Peru

**DOI:** 10.3389/fneur.2021.629257

**Published:** 2021-06-17

**Authors:** Monica M. Diaz, Marcela Gil Zacarías, Patricia Sotolongo, María F. Sanes, Donald J. Franklin, María J. Marquine, Mariana Cherner, Cesar Cárcamo, Ronald J. Ellis, Serggio Lanata, Patricia J. García

**Affiliations:** ^1^Department of Medicine, University of California, San Diego, San Diego, CA, United States; ^2^University of California Global Health Institute, San Diego, CA, United States; ^3^Facultad de Salud Pública, Universidad Peruana Cayetano Heredia, Lima, Peru; ^4^Facultad de Medicina Alberto Hurtado, Universidad Peruana Cayetano Heredia, Lima, Peru; ^5^Department of Psychology, Jackson Memorial Hospital, Miami, FL, United States; ^6^Human Immunodeficiency Virus (HIV) Neurobehavioral Research Center, University of California, San Diego, San Diego, CA, United States; ^7^Department of Psychiatry, University of California, San Diego, San Diego, CA, United States; ^8^Department of Neurosciences, University of California, San Diego, San Diego, CA, United States; ^9^Weill Institute for Neurosciences, Memory and Aging Center, University of California, San Francisco, San Francisco, CA, United States; ^10^Global Brain Health Institute, University of California, San Francisco, San Francisco, CA, United States; ^11^School of Public Health, University of Washington, Seattle, WA, United States

**Keywords:** HIV-associated neurocognitive disorder (HAND), HIV/AIDS & infectious diseases, cognitive impairment, dementia, Latin America, Peru, non-communicable disease

## Abstract

**Background:** With widespread use of antiretroviral medications, people living with HIV (PWH) are living longer worldwide, increasing their risk of developing neurocognitive impairment (NCI). The proportion of Peruvians over age 60 is expected to increase to 25% of the population by 2050, including PWH. Therefore, the problem of aging and NCI, especially in the setting of HIV infection, is uniquely pressing. We sought to study the rates of and risk factors associated with NCI among middle-aged and older PWH in Lima, Peru.

**Materials and Methods:** Sociodemographic, medical (infectious and non-infectious), and psychiatric comorbidity and laboratory data were collected. We administered a brief neuropsychological battery evaluating seven cognitive domains affected in HIV-associated NCI and a depression screening. Cognitive test raw scores were converted to *T*-scores that were demographically adjusted. Descriptive statistics were performed together with regression (unadjusted and adjusted) analyses to determine potential risk factors for NCI among PWH.

**Results:** This was a cross-sectional study in which 144 PWH aged ≥40 years attending a large HIV clinic in Lima, Peru, were recruited from September 2019 to March 2020. Mean age was 51.6 ± 7.7 years, and mean years of education were 14.0 ± 3.1 with 15% females. Median [interquartile range (IQR)] current CD4 and nadir CD4 were 554 (371, 723) and 179 (83, 291), respectively, and 10% currently had AIDS. The prevalence of NCI was 28.5%, and many demonstrated difficulty with attention and working memory (70%). One-quarter of PWH had mild depression or worse on Patient Health Questionnaire 9 (PHQ-9 ≥ 5). In bivariate analyses, neither a depression history nor a higher PHQ-9 score correlated with NCI. No other non-communicable medical or psychiatric comorbidity nor HIV characteristic was predictive of NCI. Having a positive lifetime history of hepatitis B infection, pulmonary tuberculosis, or syphilis increased risk of NCI (PR 1.72; 95% CI 1.04–2.86) in unadjusted analyses, but not in adjusted analyses.

**Conclusions:** NCI among older Peruvians with HIV was found to be highly prevalent with levels consistent with prior reports of HIV-associated NCI worldwide. Common latent HIV-associated co-infections, including latent syphilis, hepatitis B infection, or pulmonary tuberculosis, may increase the risk of NCI among middle-aged and older PWH in Peru.

## Introduction

The number of people living with dementia of any cause worldwide in 2015 was estimated at 48 million people, and this figure is expected to rise to 135 million by 2050, with 63% of cases living in low- and middle-income countries (LMICs) (1). With increased access and use of antiretroviral therapy (ART), the life expectancy of people living with HIV (PWH) has markedly increased (2, 3). This represents a breakthrough in the field but also a new challenge due to the increasing prevalence of non-communicable comorbidities, such as diabetes mellitus and hyperlipidemia, associated with aging with HIV including various forms of neurocognitive impairment (NCI) (4, 5). In addition, our understanding of risk factors for NCI has evolved over time during the ART era, with more chronic comorbidities thought to worsen NCI as PWH live longer (6–8). HIV-associated neurocognitive disorder (HAND) is the most common form of NCI among PWH. It presents with varying degrees of neurologic dysfunction (9) and is associated with increased morbidity and mortality (3, 7, 10). Since the introduction of ART, the incidence of HIV-associated dementia, a severe form of HAND, has decreased (11, 12), but the overall prevalence of HAND worldwide remains stable (7, 8). Peru is a country of 32 million people that is undergoing rapid aging. Currently, 3.3 million people are over the age of 60 (13), and it is estimated that by 2050, 25% of the Peruvian population will be over age 60 (1). Therefore, the problem of aging and NCI is uniquely pressing among PWH.

Several studies have reported that the prevalence of HAND in North America and in some European countries exceeds 30% and affects more than 50% of people with Acquired Immune Deficiency Syndrome (AIDS) (7, 8, 14, 15). There is a paucity of research on the prevalence and characterization of HAND in Latin America. Published studies have reported the prevalence of mild neurocognitive disorder, a milder symptomatic form of NCI, in PWH at 20%, and ~50% have asymptomatic NCI without functional impairment (16, 17). In Peru in particular, there are limited studies addressing NCI among PWH with no studies specifically on middle-aged to older PWH (18, 19). Moreover, there are no standard cognitive screening protocols nor clinical management guidelines for PWH with NCI despite 70,000 PWH living in Peru, with 60% currently having access to ART (20, 21). Cognitive decline in the general population presents significant medical, social, and economic challenges (22), and Peruvian health systems, like those of many Latin American countries, will face challenges with the increasing burden of NCI (1). Documenting the prevalence of HAND and associated risk factors among Peruvians with HIV is the first step in identifying the burden of this disease, which may lead to implementation of public policies that can help PWH living with NCI to improve their quality of life. In this study, we sought to study rates of HIV-related NCI in a group of older Peruvians with HIV living in Lima and determine the risk factors for NCI among PWH.

## Materials and Methods

This was a cross-sectional study of PWH living in Lima, Peru, in which demographic data were collected and clinical and neurocognitive evaluations were performed. This study was conducted from September 2019 until March 2020. Participants were men and women aged 40 years or older, Peruvian-born, and native Spanish speakers recruited from a large HIV clinic run by a non-governmental organization. All PWH enrolled had a record of a positive ELISA and Western blot tests in their medical chart and had been receiving ART therapy for at least 1 month at the outpatient clinic. All participants had completed at least 6 years of schooling (primary school) and had the ability and willingness to participate in the study and provide informed consent. Individuals with a self-reported history of non-HIV-related neuromedical comorbidities that may cause NCI were excluded by administering to potential participants a screening questionnaire prior to their enrollment. This screening questionnaire included questions about any known non-HIV-related neurological disorder that led to cognitive impairment (e.g., epilepsy or stroke), psychotic disorders (schizophrenia or bipolar disorder), and brain injury with loss of consciousness for more than 30 min without return to premorbid baseline. Those with a current and lifetime substance use disorder according to the *Diagnostic and Statistical Manual of Mental Disorders*-5 (DSM-5) were excluded.

### Study Procedures

Eligible patients attending the HIV clinic were identified by study personnel the day prior to their visit if they met the age criteria for enrollment. Nurses and physicians invited these potentially eligible patients to enroll in the study. With the patient's verbal consent, study personnel then contacted the patients by phone and invited them to the clinic for a scheduled study visit. At the study visit, all study procedures were explained to the participant with ample time to answer any questions, and written informed consent was obtained. Patients were then interviewed and examined by a neurologist (MMD) or a physician research assistant with training in neurological examination and were supervised by the neurologist. Sociodemographic information including age, sex, years of education achieved, occupation, place of birth, and place of current residence was collected. Self-reported past medical history, including history of prior infections [opportunistic (in PWH only) and non-opportunistic] and chronic non-communicable diseases, was also collected and was corroborated with review of the medical chart following the participant's study visit. We also administered an ART adherence questionnaire to all participants. All participants underwent a comprehensive neuromedical assessment including a complete neurological exam. Following the physical examination, a neurocognitive battery was administered to evaluate neurocognitive function, the Pfeffer Activities of Daily Living questionnaire (PFAQ) to determine functional status, and the Patient Health Questionnaire (PHQ)-9 to screen for depression (Section Instruments Utilized).

We obtained the following information from the medical chart: prior and current ART regimens, any adverse events related to ART, corroboration of self-reported past medical history with physician's clinic assessments in the chart. Laboratory data obtained from chart review included any lifetime positive rapid plasma reagin (RPR) test, herpes simplex virus (HSV)-1 antibody test, tuberculosis (TB) sputum test, and hepatitis B surface antigen (HbsAg) test results. We also noted the most recent hemoglobin, creatinine, total cholesterol, and triglyceride levels.

Following the study visit, the results of the neurocognitive battery were normed as described below, and participants and their treating physician were given a summary of their test results. We provided participants with recommendations on healthy living strategies and strategies for prevention of cognitive decline (23). For those in whom NCI was detected, a neurologist (MMD) counseled the participant by phone or in-person visit on their results and NCI prevention strategies, and results were discussed with the treating physician.

### Instruments Utilized

#### Neuropsychological Test Battery

The neuropsychological test battery evaluated seven cognitive domains [abstraction/executive function, motor performance, memory (learning and recall), attention/working memory, verbal fluency/language, speed of information processing, and visuospatial orientation] that are commonly affected in HIV-associated NCI and that have been widely utilized to assess HIV-associated NCI in the United States, in Europe (9, 24, 25), and in Brazil (26). The domains were evaluated using the following tests:

- Abstraction and executive function: Color Trails Test 2 (27);
- Motor performance: Grooved Pegboard (dominant hand) and Grooved Pegboard (non-dominant hand) (28);
- Memory (learning and recall): Hopkins Verbal Learning Test-Revised (HVLT-R)-Total Learning, HVLT-R Delayed Recall (29), and Benson Figure Recall (30);
- Attention and working memory: Weschler Adult Intelligence Scale (WAIS)-3 Digit Span (31);
- Verbal and language fluency: semantic/category fluency (Animal Naming) and letter fluency (PMR) (32);
- Speed of information processing: Color Trails Test 1 (33); and
- Visuospatial orientation: Benson Figure immediate copy (34).

The instruments have been translated, and most have been validated into Spanish by native Spanish speakers and used in several other studies for Spanish speakers in Latin America (35–37). Raw test scores were converted into demographically adjusted *T*-scores (adjusted for age, sex, and education level) for each test. Norms for native Spanish speakers from the Neuropsychological Norms for the US-Mexico Border Region in Spanish (NP-NUMBRS) were applied to all tests when available, including for semantic/category fluency (Animal Naming) (38), letter fluency (PMR) (38), HVLT-R Total Learning and Delayed Recall (39), and Grooved Pegboard (dominant and non-dominant hand) (40). English-speaking norms with similar mean educational levels to that of our study were utilized for those tests for which there were no demographically adjusted Spanish NP-NUMBRS norms available given there are no Spanish-speaker norms for a Peruvian population that were adequate for our population.

Normed *T*-scores were computed for each test, with regression-based adjustments for the effects of age, sex, and educational level. For each test, *T*-scores were converted to deficit scores as follows: *T* > 39 (no worse than −1 standard deviation) was considered normal and assigned a deficit score of 0. *T*-scores below 40 were converted to deficit scores as follows: 35–39 = 1 (mild impairment); 30–34 = 2 (mild to moderate impairment); 25–29 = 3 (moderate impairment); 20–24 = 4 (moderate to severe impairment); and *T* < 20 = 5 (severe impairment). For each domain, an average of the *T*-scores for each test comprising each domain were computed, and this generated a mean *T*-score for each domain. Deficit scores were summed across the test battery and then divided by the total number of individual measures to compute the Global Deficit Score (GDS), a measure of global cognitive impairment. The GDS summarizes the number and severity of neurocognitive deficits across the entire test battery. A GDS cutoff of ≥0.50 was used to determine global NCI (7, 41, 42).

#### Pfeffer Activities of Daily Living Questionnaire (PFAQ)

Subjective cognitive difficulties were assessed using the validated Spanish version of PFAQ (43–45). In the PFAQ, participants rate themselves as having or not having cognitive difficulties in their daily lives on a 4-point scale, in domains of memory, language and communication, sensory perception, motor skills, and higher-level cognitive functions. The score used is the sum of items on which the participants reported experiencing difficulties ranging from normal (0) to dependent (3), for a total of 30 points, with higher scores indicating worse functional status (43). When a caregiver or companion was present during the interview, the questionnaire was corroborated with the caregivers' report with the participants' permission. Employment status was derived from the demographic interview, which collected information on whether the participant was working and the type of employment (Supplementary Material 1).

#### PHQ-9 for Depression Screening

This is a nine-item questionnaire designed to screen for depression in primary care and other medical settings was administered to all participants as a depression screening measure (46–48). The PHQ-9 scores each of the nine DSM-IV criteria for depression as “0” (not at all) to “3” (nearly every day), addressing somatic (fatigue, appetite, and sleep quality) and non-somatic (suicidal ideation and feelings of guilt) depressive symptoms; higher scores indicate worse depressive symptomatology. The standard cutoff score to identify major depression is 10 or above (46–48). The PHQ-9 has been previously validated in Spanish for use in Peru (49), has been used in other Spanish-speaking populations throughout Latin America and Spain (50–52), and has been validated for use in depression screening in HIV in South Africa (53) (Supplementary Material 2).

### NCI (or HAND) Diagnosis for PWH

Global cognitive impairment was defined as a GDS score ≥0.5, and individual domain cognitive impairment was defined as a domain-averaged T-score <40. HIV-associated NCI diagnoses were assigned according to the Frascati criteria (9). To receive a diagnosis of HIV-associated dementia, participants had to have moderate to severe impairment on neuropsychological testing and require major assistance in activities of daily living based on the PFAQ. Mild neurocognitive disorder was diagnosed when NCI was mild to moderate on neuropsychological testing by GDS score, and difficulties were reported in two Pfeffer areas except that for participants with at least moderate depressive symptomatology on the PHQ-9; three areas were required on the Pfeffer questionnaire. Asymptomatic NCI was defined as mild to moderate impairment without any functional impairment on the Pfeffer questionnaire.

### Statistical Analyses

Descriptive statistics were computed with means [standard deviations (SDs)] or medians [interquartile ranges (IQRs)] for all demographic and HIV characteristic continuous variables. Frequencies and percentages were computed for past medical and psychiatric history variables, and to determine the frequency of depressive symptoms, functional dependence and cognitive impairment were used based on defined cutoff points for each test. Univariable (without adjustment for covariates) and multivariable (adjusted for relevant covariates) regression analyses were performed using GLM with link log and family Poisson to obtain unadjusted and adjusted prevalence ratios (PR and aPR, respectively). All covariates [age ≥50 years, female sex, educational level of secondary school or less, comorbid conditions (hypertension, hyperlipidemia, anemia, self-reported depression, PHQ-9 score ≥ 5, self-reported anxiety, past hepatitis B infection, past TB infection, and past syphilis infection)], current absolute CD4 count <500, nadir CD4 count 51–200, nadir CD4 count <50, detectable plasma viral load, and HIV duration ≥5 years) with a *p* < 0.10 in univariable analyses were included as covariates in the multivariable analyses. A *p* < 0.05 was considered statistically significant. All statistical analyses were performed using the JMP Pro^®^ statistical software, version 14.2.0 (SAS Institute Inc., Cary, NC, USA) and STATA (College Station, TX, USA).

### Ethical and Institutional Review Board Approvals

The study and instruments were approved by the institutional review boards of Universidad Peruana Cayetano Heredia (Lima, Peru) and Via Libre (Lima, Peru). The institutional review board of the University of California, San Diego (San Diego, CA, USA), exempted the study from review. Written informed consent was obtained from study participants once the research procedure was explained to them, and sufficient time was given for participants to have any questions answered. Participants were not reimbursed for their time.

## Results

### Demographics and Medical Characteristics

We recruited 144 PWH with a mean age of 51.6 ± 7.7 years and mean years of education of 14.0 ± 3.1 (15.2% females). We found that 2/144 (1.4%) have completed up to primary school, 48/144 (33.3%) have completed up to secondary school, 76/144 (52.8%) have had some or completed university or technical school, and 18/144 (12.5%) have completed a post-graduate degree (data not shown). We found that a small proportion of PWH were unemployed (13.0%) (Table 1).

**Table 1:** Demographic and medical characteristics (*N* = 144).

	**Median [IQR], Mean (SD), or n (%)**
**Demographic variables**	
Age	51.6 (7.7)
Sex (*n*, % females)	22 (15.2%)
Education (years)	14.0 (3.1)
Unemployed or retired	18 (13.0%)
**Past non-infectious medical or psychiatric history**^**a**^	
Hypertension	19 (13.7%)
Hyperlipidemia	46 (32.6%)
Diabetes or prediabetes	7 (5%)
Anemia	14 (10%)
Seizure (ever)	6 (4.3%)
Depression (by self-report)	21 (15.2%)
PHQ-9 total score	3.34 (4.10)
Depression *(PHQ-9 > 4)*	35 (25.2%)
Anxiety (by self-report)	15 (10.9%)
**Past infectious medical history**^**a**^	
Pulmonary TB	21 (15.2%)
Completed TB treatment	21 (100%)
Herpes simplex virus	43 (30.9%)
Syphilis	41 (29.5%)
Completed syphilis treatment	35/37 (97.2%)
Any hepatitis type	50 (35.7%)
Hepatitis A	26 (18.1%)
Hepatitis B	24 (16.7%)
Hepatitis C	2 (1.4%)
CNS infection	5 (3.6%)
*CNS amoebiasis*	1 (20%)
*Cryptococcal meningitis*	1 (20%)
*Herpes encephalitis*	1 (20%)
*Neurocysticercosis*	1 (20%)
*CNS toxoplasmosis*	1 (20%)
**Current substance use**^**a**^	
Alcohol use	68 (47%)
Cigarette smoking	24 (16.7%)
Marijuana use	4 (2.8%)
Cocaine use	2 (1.4%)

a *by self-report and corroboration with medical chart whenever possible*.

*IQR, interquartile range; SD, standard deviation; TB, tuberculosis*.

Non-communicable comorbidities were common. Less than one third had dyslipidemia (32.6%), 13.7% had hypertension, and very few had diabetes (5%), anemia (10%), or lifetime seizure history (4.3%) (Table 1). The most recent mean hemoglobin reported in laboratory analyses was a mean (SD) of 13.9 (1.52), and mean creatinine levels were within the normal range [0.93 (0.17)]. Recent mean total cholesterol levels were 196.2 (46.0), and triglycerides were 170.7 (98.1) (Table 2). Alcohol use was more common among PWH (47%), but current cigarette smoking (16.7%), marijuana use (2.8%), and cocaine use (1.4%) were less common (Table 1). Self-reported past infectious history (corroborated with chart review) was obtained. Past history of pulmonary TB (15.2%), HSV infection (30.9%), and syphilis (29.5%) was common. All participants with a positive TB history completed treatment, and nearly all who had a history of syphilis per self-report completed treatment (97.2% of those with a syphilis history). Of those participants who had an RPR test in the medical chart, 28/105 (26.7%) had a positive RPR test in the past. Of 92 participants, eight (8.7%) had a positive lifetime TB sputum test (Table 2). Prior hepatitis infection was common among PWH with more than one third (35.7%) having had any hepatitis infection in the past (hepatitis A 18.1%, hepatitis B 16.7%, and hepatitis C 1.4%) (Table 1). Of those who had an HBsAg test available in their medical chart, 11/100 (11%) had a positive HBsAg. There were five cases of a prior central nervous system (CNS) infection by self-report, including CNS amoebiasis, cryptococcal meningitis, HSV encephalitis, neurocysticercosis, and CNS toxoplasmosis reported (Table 1).

**Table 2:** HIV characteristics and laboratory results (*N* = 144).

	**Median [IQR], Mean (SD),**
	**or n (%)**
**HIV characteristics**	
Current CD4^a^	554 [372–723]
Nadir CD4^b^	179 [83–261]
AIDS history	
HIV infection duration (years)	9.9 (7.1)
On antiretrovirals	144 (100%)
Detectable plasma viral load (>50 copies/mL)	18/126 (14.3%)
**Current antiretroviral use (*****n=*** **131)**	
Nucleoside reverse transcriptase inhibitor (NRTI)	
Lamivudine (3TC)	80 (61.1%)
Tenofovir (TDF)	80 (61.1%)
Emtricitabine (FTC)	45 (34.4%)
Zidovudine (AZT)	36 (27.5%)
Abacavir (ABC)	12 (9.2%)
Stavudine (D4T)	1 (0.7%)
Didanosine	0 (0%)
Non-nucleoside reverse transcriptase inhibitors (NNRTI)	
Efavirenz (EFV)	91 (69.5%)
Nevirapine (NVP)	15 (11.5%)
Protease inhibitor	
Lopinavir/ritonavir (Lop/r)	10 (7.6%)
Atazanavir (ATZ)/ritonavir	7 (5.3%)
Darunavir	2 (1.5%)
Integrase inhibitor	
Raltegravir	1 (0.8%)
Dolutegravir	1 (0.8%)
**Laboratory analyses (most recent)**	
Hemoglobin	13.9 (1.52)
Creatinine	0.93 (0.17)
Total cholesterol	196.2 (46.0)
Triglycerides	170.7 (98.1)
Positive RPR (ever)	28/105 (26.7%)
Hepatitis B surface antigen (ever)	11/100 (11%)
Positive TB sputum test (ever)	8/92 (8.7%)
Any latent HIV coinfection^c^	45/144 (31.3%)

a *of 128 participants*;

b *of 120 participants*;

c *hepatitis B, tuberculosis, or syphilis prior infections*.

*IQR, interquartile range; SD, standard deviation*.

### HIV Characteristics

PWH had a mean duration of HIV infection of 9.9 ± 7.1 years, and all participants were currently on ART. On the antiretroviral adherence questionnaire, the large majority of PWH [116/138 (84.1%)] never missed a dose in the past month, and 17/138 (12.3%) missed one to three doses in the past month, and 5/138 (3.6%) missed more than three doses in the past month. PWH had well-controlled HIV infection with a median absolute CD4 count of 554 cells/mm^3^ (IQR: 372–732) and a nadir CD4 count of 179 cells/mm^3^ (IQR: 83–291). A detectable plasma viral load (>50,000 copies/mm^3^) was noted in 14.3% of PWH. The most common non-nucleoside reverse transcriptase inhibitor (NRTI) in current use by the participants were lamivudine [3TC, 80/131 (61.1%)] and tenofovir [TDF, 80/131 (61.1%)]. Of the non-NRTIs, efavirenz was common [91/131 (69.5%)]. Protease inhibitors and integrase inhibitors were rarely used (Table 2).

### Depression and Functional Assessment Screening Results

Of the self-reported psychiatric history, 15.2% of participants self-reported a current depression diagnosis and 10.9% self-reported anxiety. Depression on the PHQ-9 depression screening (defined as PHQ-9 score ≥5) was common, with one quarter of participants screening positive for depression (Table 1). To assess functional status, a PFAQ cutoff of ≥9 was used, and we found that there were no participants that met criteria for functional dependence. When the cutoff was set lower (PFAQ total score ≥4), we found that very few had difficulty with independent activities of daily living (2.8%). In unadjusted regression analyses, only self-reported anxiety was a risk factor for depression on PHQ-9 [PR 2.03 (1.08–3.82); *p* = 0.047], but no other demographic, past medical history, or HIV characteristic variable posed an increased risk on depression by PHQ-9 score (data not shown).

### Neurocognitive Testing Results

**Figure 1:**
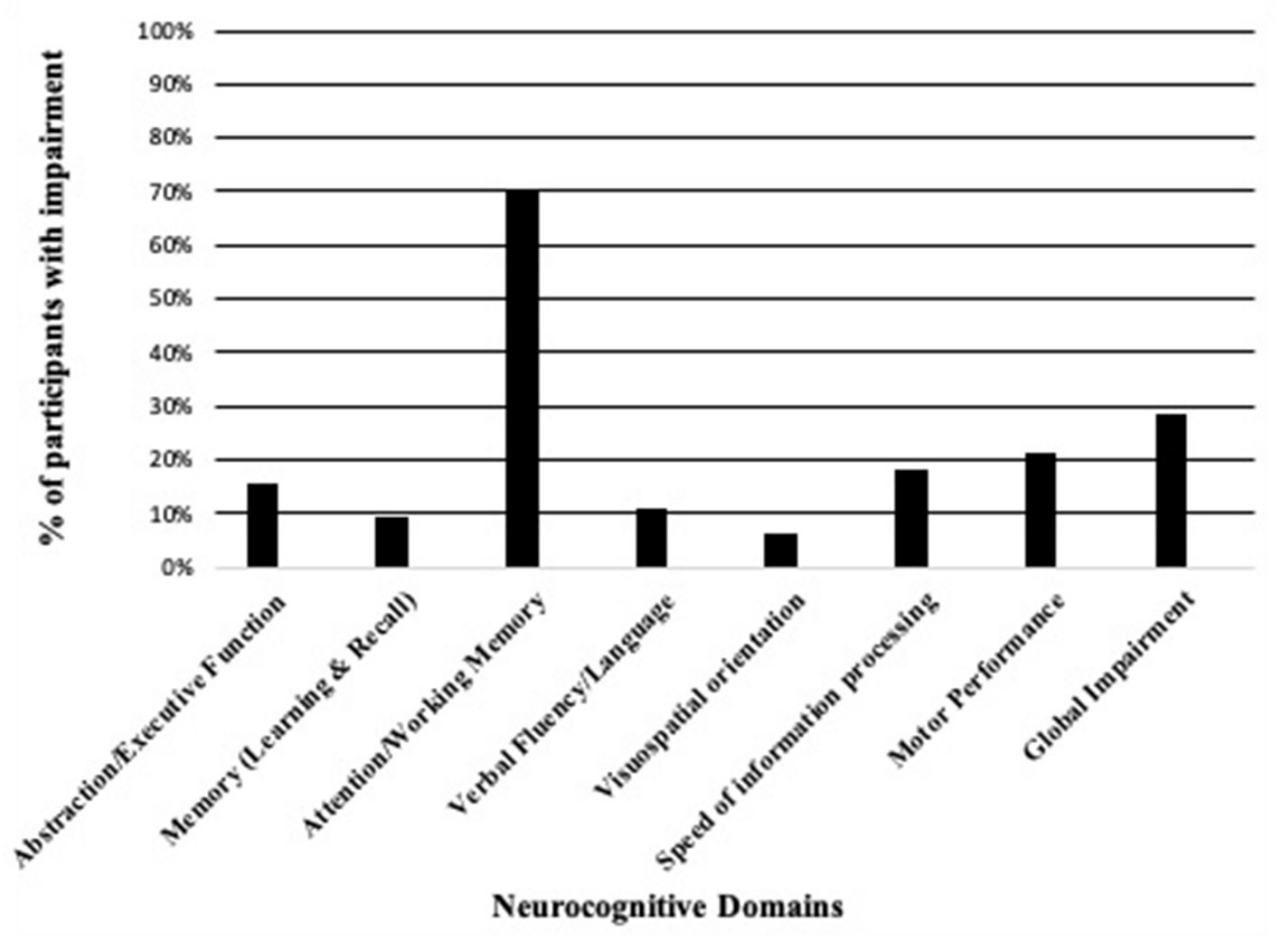
Neurocognitive domain impairment among PWH (*N* = 144).

We found that nearly 30% of participants had global NCI and that all participants with global NCI had asymptomatic NCI with no participants having mild neurocognitive disorder or HIV-associated dementia. We found high rates of impairment in the attention and working memory domain (69.9%) and that nearly one fifth of PWH (18.1%) had difficulty with speed of information processing (Figure 1, Table 3). In unadjusted regression models, HIV infection did not increase risk of NCI [PR 0.99 (0.60–1.63)], but any latent HIV coinfection (including past history of hepatitis B infection, TB infection, or syphilis) increased the risk of NCI [PR 1.72 (1.04–2.86)], and a current CD4 absolute count of <500 cells/mm^3^ approached statistical significance [PR 1.64 (0.96–2.8)]. Neither was a statistically significant risk factor associated with NCI in adjusted models [aPR 1.55 (0.81–2.92) for latent coinfection and aPR 1.64 (0.87–3.10) for absolute CD4 count <500 cells] (Table 4). In a sub-analysis of PWH (not shown) with an undetectable plasma viral load only (*n* = 126), we found that 28.6% of PWH had NCI, and in the unadjusted regression analyses, the PR of HIV positivity on NCI remained unchanged.

**Table 3:** Neurocognitive and functional status screening results of PWH (*N* = 144).

	**Median [IQR], Mean (SD), or n (%)**
**Functional status screening (PFAQ)**	
PFAQ total score	0.46 (1.10)
Functionally dependent *(PFAQ* ≥ *9)*	0 (0%)
Some difficulty with IADLs *(PFAQ* ≥ *4)*	4 (2.8%)
**Neurocognitive test results by cognitive domain (% cognitively impaired)**	
Abstraction and executive function	22 (15.3%)
Memory (learning and recall)	13 (9.4%)
Attention and working memory	100 (69.9%)
Verbal fluency and language	15 (10.5%)
Visuospatial orientation	9 (6.3%)
Speed of information processing	26 (18.1%)
Motor	30 (21.0%)
Global impairment^*^	41 (28.5%)
*Asymptomatic neurocognitive impairment*	41 (100%)
*Mild neurocognitive disorder*	0 (0%)
*HIV-associated dementia*	0 (0%)
**Neurocognitive test results by cognitive domain (demographically adjusted T-score, mean** **±** **SD)**	
Abstraction and executive function	50.9 (13.8)
Memory (learning and recall)	50.1 (7.9)
Attention and working memory	36.5 (7.6)
Verbal fluency and language	53.6 (11.4)
Visuospatial orientation	51.3 (7.0)
Speed of information processing	48.6 (14.2)
Motor	47.3 (8.8)
GDS	0.42 (0.43)

* *Global impairment considered a GDS of ≥0.5*.

*IADL, independent activities of daily living; IQR, interquartile range; PFAQ, Pfeffer Functional Assessment Questionnaire; SD, standard deviation*.

**Table 4:** Regression models (unadjusted and adjusted) for risk factors for neurocognitive impairment (*N* = 144).

	Global neurocognitive impairment^**^	
	**Yes**, ***n*** **=** **41 (28.5%)**	
**Variable**	**Unadjusted Prevalence Ratio (95% CI)**	**Adjusted Prevalence Ratio (95% CI)**
≥Age 50 years	1.27 (0.74–2.19)	–
Female sex	1.14 (0.58–2.24)	**–**
Educational level, secondary school or less	1.21 (0.72–2.03)	–
Hypertension	0.88 (0.40–1.95)	–
Hyperlipidemia	0.85 (0.48–1.52)	–
Anemia	1.24 (0.59–2.64)	–
Depression (self-report)	1.62 (0.91–2.89)	–
Depression (PHQ-9 ≥ 5)	1.54 (0.92–2.59)	–
Anxiety (self-report)	0.91 (0.38–2.20)	–
Current absolute CD4 count<500 (cells/mm^3^)	1.64 (0.96–2.8)^*^	1.64 (0.87–3.10)
Nadir CD4 51–200 (cells/mm^3^)	1.13 (0.64–1.99)	–
Nadir CD4 <50 (cells/mm^3^)	1.68 (0.91–3.09)	–
Plasma VL detectable	0.94 (0.42–2.09)	–
HIV duration ≥ 5 years	1.17 (0.65–2.08)	–
Past hepatitis B infection	1.03 (0.52–2.05)	–
Past TB infection	1.67 (0.93–3.00)	–
Past syphilis infection	0.99 (0.56–1.74)	–
Any latent HIV coinfection (hepatitis B, TB, syphilis)	**1.72 (1.04–2.86)**^**¥**^	1.55 (0.81–2.92)

* *p < 0.10*;

¥ *p < 0.05*;

** *GDS ≥ 0.5*.

## Discussion

In this study, we characterized NCI rates among middle-aged and older PWH living in Lima, Peru, and risk factors for NCI in this population. We found that nearly 30% of PWH had asymptomatic NCI and that no PWH had symptomatic NCI with functional impairment. Among PWH, a risk factor for NCI that was identified was having had a history of latent coinfection, but this was no longer statistically significant in adjusted analyses. Our findings are comparable to previously published findings with comparable NCI rates worldwide.

Several studies have assessed NCI and dementia in geriatric populations in Peru, but none have focused on an older Peruvian population with HIV. In Latin America, eight publications have described HAND prevalence and associated risk factors among PWH aged ≥40 years [seven from Brazil (16, 54–60) one from Mexico (61)], with none from Peru. These studies utilized different neurocognitive screening tools, including the International HIV Dementia Scale (IHDS) (16, 56–58) with HAND prevalence ranging from 37 to 64% using this scale or the Mini Mental State Exam (MMSE) (62) with prevalence ranging from 27 to 37%. (54, 59) Several studies from Brazil reported NCI prevalence specifically among an older age group of PWH aged ≥50 years with prevalence ranging from 23 to 54%, (16, 54, 57, 59) and in Mexico, a higher prevalence of HAND (66%) among PWH aged ≥50 years was associated with pre-frailty (61). Notably, few studies reported on asymptomatic NCI without impairment in functional status. Among Brazilian PWH with mean age of 42.5 ± 9.1 years, asymptomatic NCI rates were higher than those found in our study (48.3% in the Brazilian study vs. 28.5% in our study) (26, 63). Despite these assessments of HAND from some Latin American countries, prevalence differs depending on the geographic population surveyed and the NCI screening tool utilized and the clinical and demographic characteristics of the group, including immunosuppression, viral suppression, comorbidities, and age.

Only two studies to date have investigated NCI in HIV in Peru. One study utilized a subjective memory complaint questionnaire to determine its utility in identifying NCI in PWH in Peru; however, this sample consisted of PWH who were younger than that of our group (mean age 34.3 years) with a high proportion of alcohol use disorder (41%) and depression (42.5%) (18). Unlike in our study, this study did not assess NCI using objective standardized neurocognitive tests, limiting its utility in objective identification of NCI (18). Another international multisite study from the AIDS Clinical Trials Group included 62 ART-naïve Peruvian PWH (median age 33 and median educational level 12.5 years) (19). Although results were not reported for the Peruvian group, the study reported that across the entire international study, 19% of their population had mild NCI (19). Although the topic of NCI in PWH has been touched on in Peru, it has not been thoroughly studied in an objective manner. Our study is the first to study NCI in a group of middle-aged and older PWH from Peru using an objective multidomain neuropsychological battery.

When comparing our study results to similar studies based in the United States, we found that rates of NCI among PWH in Peru appear to be lower overall. For example, in a multisite study of 1,555 HIV+ adults, 52% of the total sample had NCI (33% of these were asymptomatic NCI, 12% mild neurocognitive disorder, and only 2% with HIV-associated dementia) (7). In another study, Latinos living in the United States had higher rates of NCI compared with non-Latino White PWH (54 vs. 42%), and Latinos tended to perform worse in speed of information processing, working memory, recall, learning, and executive function cognitive domains compared with non-Latino White PWH (37). Latinos living in the United States are heterogeneous from differing racial groups and nationalities and tend to present with worse HIV characteristics (i.e., lower nadir CD4) or non-communicable comorbidities compared with non-Latino Whites, which may worsen NCI (36, 37). In one study investigating differences in rates of NCI between US Latino and non-Latino Whites, even after adjusting for lower nadir CD4 and other HIV characteristics, Latino PWH in the United States had higher rates of NCI compared with non-Latino White PWH (OR 1.59, CI = 1.13–2.23, *p* < 0.01) (37). Therefore, it is important to consider not only common risk factors and social determinants of health that affect Latino populations with HIV in both Latin America and the United States but also the type of neurocognitive testing norms developed for these populations.

As described earlier, prior studies on HIV-associated NCI in Latin America have demonstrated that NCI and HAND prevalence differs by country depending on the screening instrument utilized. Thus, optimization of an NCI screening instrument validated against a complete neuropsychological battery, the gold standard for diagnosis of HAND (64), is needed but remains a logistical challenge. One study from Brazil, for example, compared the IHDS scale to a brief neurocognitive battery and found that the sensitivity for detection of HAND using standardized cut points of the IHDS was 36% with a specificity of 75%. The top two combinations of neuropsychological tests with the highest sensitivity compared with a gold standard neuropsychological battery were the Trail Making Test A, WAIS-3 Digit Symbol, and HVLT-R Total Recall (sensitivity 91% and specificity 96%) (63). The neuropsychological battery used in the present study included two of these three tests (WAIS-3 Digit Symbol and HVLT-R Total Recall) but replaced the Trail Making Test A with Color Trails Test 1 given a possible preemptive concern of lower literacy in our population. Although our study utilized similar tests to those in the Brazilian study, we did not compare our findings to a gold standard neuropsychological battery; thus, we are unable to report the sensitivity and specificity for detection of HAND of our brief neuropsychological battery. The tests utilized in our battery have been shown to have high sensitivity and specificity in other Brazilian studies with similar sociocultural norms and, thus, may be applicable in our population but may still have limitations (19, 63). Brazil is a Portuguese-speaking country with linguistic factors that may not be generalizable to neurocognitive testing across Spanish-speaking countries in Latin America; therefore, development of a neurocognitive battery specific to each region of Latin America is essential to capture linguistic and sociocultural factors of that region.

We found that few norms have been developed for Spanish speakers living in Peru for the majority of tests we administered; thus, we applied norms for Spanish speakers living in the United States–Mexico border with a similar demographic and sociocultural characteristics and applied norms for English speakers living in the United States when Spanish-speaker norms were unavailable for particular tests. In most prior studies on NCI, applying norms for English speakers or norms collected in Spanish-speaking countries, such as Mexico or Spain, is currently standard clinical practice (65). However, utilizing norms that are not specific to the geographic region of interest may increase the chances of either underclassifying or overclassifying NCI without appropriate demographic adjustment for that region (66). The development of neuropsychological test norms that are representative of the diversity of Latin American populations is urgently needed. This is a key first step toward the development of validated brief cognitive screening tools that can be used in routine clinical HIV care in Lima and other Latin American cities (8, 67).

Risk factors for NCI in PWH are important to consider. Some studies have found that low nadir CD4 and high plasma viral load are strong predictors of NCI among PWH on ART (7). Among ART-naïve PWH, even after treatment for a mean duration of 63 months, NCI persisted among 62.8% of those with NCI prior to treatment (25); like in our study, this study found that HIV characteristics previously associated with NCI in PWH, such as absolute CD4 cell count, plasma viral load, and use of CNS-penetrating drugs, were not associated with persistent neurocognitive deficits; however, the only risk factor that contributed to persistent neurocognitive deficits after initiation of ART was lower educational levels, highlighting the importance of educational level achieved when considering NCI evaluation (25). Our sample was largely educated with 97% of the group having completed at least secondary school, higher than the mean educational level of the general population of Peru (64% female and 75.4% male with at least some secondary schooling) (68). Two thirds of the participants in our study had some university or technical school or a post-graduate degree, likely because study recruitment took place in a specialized multidisciplinary private HIV clinic run by a non-governmental organization, and not a governmental hospital, thus attracting employed patients with higher educational levels. Given the lack of low educational levels in our sample, we did not see an association between educational levels and NCI, which may not be true of rural populations with lower educational levels in Peru. We found no other demographic variable; past medical or psychiatric history increased the risk of NCI. However, having had a latent coinfection (either hepatitis B infection, pulmonary TB, or syphilis) was a risk factor for NCI, but these infections individually did not contribute to cognitive impairment.

Latent coinfections have been described in the literature to contribute to NCI in PWH. According to the World Health Organization Global Tuberculosis report, the incidence of TB/HIV coinfection in Peru is high, with 119 reported cases per 100,000 inhabitants in the general Peruvian population and 7.5 per 100,000 among PWH in Peru (69). One of the most commonly reported active or latent HIV coinfections to worsen NCI is TB. For example, one multinational study that included patients with HIV and active TB found that participants with active TB and HIV performed statistically significantly worse on Grooved Pegboard (motor performance) and finger tapping with the non-dominant hand compared with an HIV+ non-TB group, but sustained ART for 3 years improved cognitive function among ART-naïve PWH (70). Another study from South Africa also found that active multidrug-resistant TB coinfection was associated with significantly lower domain scores in visual attention and task switching (Trail Making Test parts A and B), visual–spatial orientation and executive function (Rey Complex Figure recall), and motor performance (Grooved Pegboard Test) (71), and a similar study found that the prevalence of HAND among PWH with active multidrug-resistant TB infection was 43.5% (72). The elevated risk of cognitive impairment may be mediated by higher levels of systemic inflammation among those with coinfection as was demonstrated in a study from Zambia, which reported a prevalence of NCI among 55% of HIV+/TB patients, 34% HIV+ non-TB patients, and 14% of HIV-negative controls (73). Worse domain impairment among the HIV+ active TB group (compared with the HIV+ non-TB group) was noted in learning and memory (immediate and delayed recall), working memory, verbal fluency, and speed of information processing, but there were no differences in motor performance and executive function unlike prior studies from South Africa (72, 73). One study from southern India analyzed the effect of latent TB coinfection on NCI and found no difference in NCI prevalence between the HIV+ latent TB group and the HIV+ non-latent TB group; however, it did find that certain inflammatory markers were higher in the HIV+ latent TB group (74). Similar to the findings of this study, our study did not find a relationship between latent TB/HIV+ coinfection on NCI, but the risk of NCI increased when several coinfections were considered simultaneously.

Syphilis is another common coinfection in PWH, and prior syphilis infection was found to be common in our group of PWH (29.5%) but did not independently increase risk of NCI in PWH in our study. The prevalence of syphilis in the Peruvian population is unknown, and the country does not have a government-sponsored syphilis monitoring program; however, in one study, the prevalence of syphilis in Peru was estimated to be 0.5% among men and 0.4% among women (75). Other studies have found that syphilis coinfection may worsen NCI, including one study from the CNS HIV Anti-Retroviral Therapy Effects Research (CHARTER) cohort, which found that those with prior syphilis infection had a greater number of impaired neuropsychological test domains and a worse GDS score [0.47 (0.46) vs. 0.31 (0.33) in HIV+ non-syphilis patients, *p* = 0.03] (76). Another study demonstrated similar findings with worse neurocognitive performance among PWH with a prior history of syphilis infection; however, this group hypothesized that poor neurocognitive performance predisposes to more frequent acquisition of sexually transmitted infection due to risky behaviors (77). The relationship between syphilis and NCI is unknown, as one study demonstrated that neurosyphilis increases CNS inflammation but does not explain NCI (78). Our study did not find a relationship between past treated syphilis infection and NCI among PWH, but we did find that nearly one third of PWH in our study had syphilis infection, highlighting its prevalence in this population from Peru. Hepatitis B infection is less common compared with TB in Peru but has a prevalence of 0.75 per 100,000 inhabitants (79). Very few studies have investigated the effect of past hepatitis B infection and HIV on NCI as most studies have focused on HIV/HCV coinfection. However, in our study, hepatitis C infection was very rare, and past hepatitis B infection was much more common. Only one study found an increase in impairment of verbal learning and memory among a cohort of HCV and HBV patients without HIV compared to heathy controls (80). Our study demonstrated an increased risk of past infection with pulmonary TB, syphilis, or hepatitis B infection on NCI in unadjusted analyses, but not in adjusted analyses, highlighting the importance of management and treatment of coinfections more prevalent in Latin America to prevent NCI among PWH.

Depression risk is known to be greater in older PWH, yet few studies have reported on depression prevalence among middle-aged and older adult PWH in Latin America, particularly on its effect on NCI (81). The PHQ-9 has been utilized in Latin American populations, including in rural areas of Mexico, suggesting that the internal consistency of the PHQ-9 was good overall and in subgroups of low literacy levels, gender, and age (50). The PHQ-9 has also been validated for use in Peru (49), and studies suggest that those of lower socioeconomic status or from rural areas had lower rates of depression (82). One study from Latin America of 412 Brazilian PWH utilized the Beck Depression Inventory (BDI) for depression screening and found that a BDI score between 13 and 19 points was associated with symptomatic HAND (mild neurocognitive disorder and HIV-associated dementia) (16). Another study of older Brazilian PWH aged ≥50 years found that the frequency of mild neurocognitive disorder using the MMSE was 36.5% and that depressive symptoms were present in 34.6% of participants using the BDI-II (59). This study found that depression was a risk factor for greater functional impairment (59), yet our study results do not suggest a relationship between higher PHQ-9 score and NCI among PWH, likely because we did not identify persons with symptomatic HAND or functional impairment. A unified approach to identifying those older PWH at greatest risk for depression is needed by applying culturally appropriate depression screening tools and considering depression screening when evaluating for NCI in older PWH.

Our study has limitations. Firstly, this was a cross-sectional study capturing NCI at one isolated time point during the course of HIV illness and did not capture longitudinal data on cognitive decline. Thus, no inferences can be made on the risk of progressing from asymptomatic NCI to symptomatic forms of HAND (mild neurocognitive disorder or HIV-associated dementia). Second, there are no neuropsychological test norms specific to Lima, Peru; thus, norms for Spanish speakers with similar sociocultural norms were utilized when available (NP-NUMBRS), and English-speaking norms were used for those in which NP-NUMBRS norms were unavailable. Although we adjusted for demographic variables (age, sex, and educational level), without neuropsychological test norms specific to the region of study, NCI may be underestimated or overestimated. Lastly, it is important to note that this study and the neuropsychological battery utilized may not be generalizable to populations of lower literacy or lower educational levels, particularly in rural populations. Participants in our study had a mean educational level of 14 ± 3 years, with the large majority (97%) having completed at least secondary school and two-thirds having had some university or technical school. This figure is much greater than the proportion of Peruvians in the general population across all of Peru with up to secondary school completed (64% female and 75.4% male) (68). Our results may be generalizable to certain regions of Lima with higher educational levels comparable to our population, but they may not be generalizable to rural populations or other metropolitan areas of Peru and other LAC. Illiteracy and low educational levels are common across Latin America, including Peru, where the illiteracy level is 6% (83), and higher among older adults living in rural areas of Peru compared with urban settings (41.6% rural vs. 12.3% urban), thus limiting the external validity of our results to low-literacy or illiterate populations in Peru and elsewhere in Latin America (84). Lastly, the sample size was not large (*N* = 144), thus limiting conclusions that may be made regarding risk factors of NCI. However, a *post-hoc* power calculation was conducted to determine the power of this study to detect TB as a risk factor of interest for cognitive impairment among PWH. TB was selected as a risk factor for NCI in the *post-hoc* power calculation because of evidence from previous studies that it is a risk factor for cognitive impairment (71, 74); it is a common exposure in Peru (85) and is commonly associated with HIV (86). Given that our secondary aim was to explore risk factors related to NCI, we calculated the power to detect a statistically significant difference in the exposure (TB) when comparing its frequency among those with and without NCI. Given that 21 participants had a history of TB, our study had a power > 80% for a PR of 3.35 or higher.

Despite these limitations, our study is the first to report the rates of NCI among a group of middle-aged and older adult Peruvians with HIV living in the capital city of Lima, utilizing a multidomain objective neurocognitive battery. We have highlighted the need to validate a brief cognitive screening tool that may be generalizable and applicable across the wide spectrum of educational and literacy levels, in order to enhance the external validity of the tool. Neuropsychological test norms specific to Lima, Peru, that account for the sociocultural context and demographics of urban Peru are also key in being able to properly define the rate of NCI seen in an aging HIV population. There is a need for long-term longitudinal data that characterizes whether people with NCI without functional impairment may progress to symptomatic forms with functional impairment and whether cognitive decline should be a concern as PWH age in Peru and throughout Latin America. Demonstrating these aspects of HIV-associated NCI across all of Peru, including in urban and rural areas across the spectrum of educational levels, will allow for the creation of new therapeutic targets and management strategies for HAND in LMICs.

## Data Availability Statement

The raw data supporting the conclusions of this article will be made available by the authors, without undue reservation.

## Ethics Statement

The studies involving human participants were reviewed and approved by Universidad Peruana Cayetano Heredia (Lima, Peru). The patients/participants provided their written informed consent to participate in this study.

## Author Contributions

MD study concept and design, data collection, performance of statistical analyses, interpretation of analyses, and drafting of manuscript. MZ study concept and design, data collection, interpretation of analyses, and drafting of manuscript. PS study concept and design, analysis and interpretation, and drafting of manuscript. MS data collection, interpretation of analyses, and drafting of manuscript. DF data management and analyses and drafting of manuscript. MM and MC study concept and design and critical revision of the manuscript for important intellectual content. CC supervised statistical analyses, interpretation of analyses, and critical revision of manuscript. RE and SL study concept and design, interpretation of analyses, and critical revision of the manuscript for important intellectual content. PG study concept and design, interpretation of analyses, drafting of the manuscript, and critical revision of the manuscript for important intellectual content. All authors contributed to the article and approved the submitted version.

## Conflict of Interest

The authors declare that the research was conducted in the absence of any commercial or financial relationships that could be construed as a potential conflict of interest.
