# Supplementary material for: Characterization of HIV-Associated Neurocognitive Impairment in Middle-Aged and Older Persons With HIV in Lima, Peru
Source: Front Neurol. 2021 Jun 17;12:629257. doi: 10.3389/fneur.2021.629257 (PMC8248346; doi:10.3389/fneur.2021.629257)
Supplement: Supplementary file 1 [file Data_Sheet_1.PDF]

PHQ9P

C U E S T I O N A R I O   S O B R E   L A   S A L U D   D E L   P A C I E N T E - 9 72883

(US Spanish version of the PHQ)

**Durante las <u>últimas 2 semanas</u>, ¿qué tan seguido ha tenido molestias por cualquiera de las siguientes dificultades?**	**No del todo**	**Varios días**	**Más de la mitad de los días**	**Casi todos los días**
1. Poco interés o placer en hacer cosas	0	1	2	3
2. Sintiéndose decaído(a), deprimido(a), o sin esperanzas	0	1	2	3
3. Dificultad en caer o permanecer dormido(a), o dormir demasiado	0	1	2	3
4. Sintiéndose cansado o teniendo poca energía	0	1	2	3
5. Pobre de apetito o comer en exceso	0	1	2	3
6. Sintiéndose mal con usted mismo(a) – o que usted es un fracaso o que ha quedado mal con usted mismo(a) o con su familia	0	1	2	3
7. Dificultad en concentrarse en cosas, tales como leer el periódico o ver televisión	0	1	2	3
8. ¿Moviéndose o hablando tan lento, que otras personas podrían notarlo? O lo contrario – muy inquieto(a) o agitado(a) que usted ha estado moviéndose mucho más de lo normal	0	1	2	3
9. Pensamientos de que usted estaría mejor muerto(a) o de alguna manera lastimándose a usted mismo(a)	0	1	2	3

SCORING FOR USE BY STUDY PERSONNEL ONLY

0 + ______ + ______ + ______

=Total Score: ______

Si usted marcó <u>cualquiera</u> de los problemas, ¿qué tan <u>difícil</u> han afectado estos problemas en hacer su trabajo, encargarse de tareas del hogar, o llevarse bien con otras personas?

**Para nada difícil**	**Un poco difícil**	**Muy difícil**	**Extremadamente difícil**
☐	☐	☐	☐

Copyright © 2005 Pfizer Inc. Todos los derechos reservados. Reproducido con permiso. EPI0905.PHQ9P

Confirmo que la información en este formulario es correcta.	Iniciales del paciente:	Fecha:

____________________ MRN ______________________________________ PROVEEDOR
