# Supplementary material for: Characterization of HIV-Associated Neurocognitive Impairment in Middle-Aged and Older Persons With HIV in Lima, Peru
Source: Front Neurol. 2021 Jun 17;12:629257. doi: 10.3389/fneur.2021.629257 (PMC8248346; doi:10.3389/fneur.2021.629257)
Supplement: Supplementary file 2 [file Data_Sheet_2.DOCX]

**Cuestionario de ACTIVIDAD FUNCIONAL de PFEFFER (FAQ)**

**Pfeffer et al. (1982)**

# Informador (relación con el paciente):

Nombre: Varón [ ] Mujer [ ]

Fecha: F. nacimiento: Edad:

Estudios/Profesión: N. Hª: Observaciones:

# Puntuar cada ítem del modo siguiente:

1. ***Normal; o nunca lo hizo pero podría hacerlo solo/a***
2. ***Con dificultad pero se maneja solo; o nunca lo hizo y si tuviera que hacerlo ahora tendría dificultad***
3. ***Necesita ayuda (pero lo hace)***
4. ***Dependiente (no puede realizarlo)***

| 1. ¿Maneja su propio dinero ? | **3** | **2** | **1** | **0** |
| --- | --- | --- | --- | --- |
| 2. ¿Puede hacer solo/a la compra (alimentos, ropa, cosas de la casa)? | **3** | **2** | **1** | **0** |
| 3. ¿Puede preparase solo/a el café o el té y luego apagar el fuego? | **3** | **2** | **1** | **0** |
| 4. ¿Puede hacerse solo/a la comida? | **3** | **2** | **1** | **0** |
| 5. ¿Está al corriente de las noticias de su vecindario, de su comunidad? | **3** | **2** | **1** | **0** |
| 6. ¿Puede prestar atención, entender y discutir las noticias de la radio y los programas de TV, libros, revistas? | **3** | **2** | **1** | **0** |
| 7. ¿Recuerda si queda con alguien, las fiestas familiares (cumpleaños, aniversarios), los días festivos? | **3** | **2** | **1** | **0** |
| 8. ¿Es capaz de manejar su propia medicación? | **3** | **2** | **1** | **0** |
| 9. ¿Es capaz de viajar solo/a fuera de su barrio y volver a casa? | **3** | **2** | **1** | **0** |
| 10. ¿Saluda apropiadamente a sus amistades? | **3** | **2** | **1** | **0** |
| 11. ¿Puede salir a la calle solo/a sin peligro? | **3** | **2** | **1** | **0** |
| **PUNTUACIÓN TOTAL** |  | | | |

Una puntuación por debajo de 6 indica normalidad (no dependencia) Una puntuación de 6 o más indica alteración funcional
